# Prevalence of adenovirus in children with acute respiratory tract infection in Lanzhou, China

**DOI:** 10.1186/1743-422X-10-271

**Published:** 2013-08-29

**Authors:** Yu Jin, Rong-fang Zhang, Zhi-ping Xie, Kun-long Yan, Han-chun Gao, Jing-rong Song, Xin-hui Yuan, Yun-de Hou, Zhao-jun Duan

**Affiliations:** 1 Medical School of Nanjing University, Nanjing, 210008, China; 2 Nanjing Children’s Hospital, Nanjing, 210008, China; 3 Gansu provincial maternity and child-care hospital, Gansu 730050, China; 4 Key Laboratory for Medical Virology, Ministry of Health, National Institute for Viral Disease Control and Prevention, China CDC, Beijing 100052, China

**Keywords:** Adenovirus, Respiratory tract infection, PCR, Children

## Abstract

**Background:**

Human adenovirus (HAdV) is an important agent causing respiratory tract infection in children. Information on the epidemiological and clinical features of HAdV is limited in children with acute respiratory tract infections (ARTIs) in China, especially those of a novel genotype, Ad55.

**Methods:**

In total, 1169 nasopharyngeal aspirates were collected from children younger than 14 years with ARTIs between November 2006 and November 2009. The polymerase chain reaction (PCR) was used to screen HAdVs. All PCR-positive products were sequenced.

**Results:**

74 of 1169 (6.33%) specimens were positive for HAdVs. Among positive cases, AdV3 (58/74) was detected most frequently, followed by AdV11 (10/74), AdV2 (2/74), AdV7 (2/69), AdV6 (1/74), and AdV1 (1/74). AdV55 was found in one case. The incidence of HAdV infection peaked in children aged 3–7 years. The most common clinical diagnosis was upper respiratory infection, and the most common syndrome was fever and cough.The comparison of HAdV and RSV group revealed that Children infected with group AdV were significant older than children infected with group RSV, had more fever but less frequently wheezing, and cough, crackles, and cyanosis, The duration of hospitalization between the AdV group and RSV group was not significant, but a greater frequency of LRTIs was observed in RSV group.

**Conclusions:**

HAdV is an important viral agent in children with ARTIs in Lanzhou City, China. Multiple HAdV serotypes co-circulated with Ad3, which was predominant in this 3-year study. The novel AdV55 genotype was found in one case. No fixed seasonal rhythm could be identified.

## Introduction

Acute respiratory tract infections (ARTIs) are a major health problem worldwide, with high morbidity and mortality rates. Human adenovirus (HAdV) is not only an important cause of mild upper respiratory tract illness, but is also associated with more serious diseases, such as severe pneumonia. HAdV is responsible for 5–10% of lower respiratory tract infection (LRTI) in children [1,2]. Additionally, the occurrence of fatal outcomes and chronic pulmonary sequelae associated with HAdV infection has been reported frequently [3-5].

HAdV infections can occur endemically throughout the year or in epidemics. In most studies, HAdV have been isolated endemically throughout the year [1,6,7], but numerous outbreaks of ARTI caused by HAdV have been reported during the last decade in many countries. Particular species and serotypes are more commonly associated with disease, syndromes, epidemiological settings, and demographic risk groups [8]; however, the prevalence of HAdV in Lanzhou remains unknow.

HAdV belongs to the *Mastadenovirus* genus and consists of seven species (A–G). To date, 67 types have been identified[8-13]. Whether these new types represent truly new strains or simply recombination events without clinical significance, needs to be further investigated. Respiratory diseases are generally associated with species B (serotypes 3 and 7), C (serotypes 1, 2, and 5), and E (serotype 4) viruses, whereas viruses in species F cause gastroenteritis, and viruses in subspecies D are often associated with keratoconjunctivitis. A study from Cuba recently reported that species D can also be isolated in respiratory specimens as a dominant genotype [14]. In China, AdV3 and AdV7 have been reported as causative agents in epidemic outbreaks of respiratory disease. In 2009, a study from the Chinese Centers for Disease Control (CDC) reported an HAdV acute respiratory disease outbreak in Qishan, Shanxi Province, and AdV55 (QS-DLL), a new adenovirus not previously reported worldwide, was identified as the cause of these infections. The novel HAdV was shown to arise from a hexon recombination between AdV11 and AdV14 [11]. This outbreak of AdV55 in China prompted us to review HAdV-associated ARTIs in Lanzhou City, China.

The objective of this study was to describe the epidemiological and clinical features of HAdV ARTIs over a period of 3 years.

## Materials and methods

### Study participants and samples

Between November 2006 and November 2009, 1169 nasopharyngeal aspirates (NPAs), consisting of 764 inpatient specimens and 405 outpatient specimens, were collected from 1169 children with ARTIs in the First Hospital of Lanzhou University, Gansu Province, China.

Informed consent was obtained from the parents of all children who provided specimens. The study protocol was approved by the hospital’s ethics committee (The Ethics Committee of the First Hospital of Lanzhou University).

All NPA specimens were collected and transported immediately to the laboratory at the National Institute for Viral Disease Control and Prevention, China CDC, and stored at -80°C until further analysis. Demographic data and clinical findings were recorded.

### DNA/RNA extraction

Viral DNA and RNA were extracted from 140 μL of each NPA specimen using the QIAamp viral DNA and RNA mini kits (Qiagen, Shanghai, China) according to the manufacturer’s protocol. cDNA was synthesized using random hexamer primers with Superscript II RH^-^ reverse transcriptase (Invitrogen, Carlsbad, CA, USA).

### HAdVs detection

In screening for HAdV, we used forward (TTCCCCATGGCICAYAACAC) and reverse (CCCTGGTAKCCRATRTTGTA) primers that target the partial region of the hexon gene to amplify a 482-bp fragment, as described elsewhere [15]. The polymerase chain reaction (PCR) was performed under the following conditions: 94°C for 8 min, followed by 35 cycles at 94°C for 30 s, 50°C for 30 s, and 72°C for 45 s, and a final extension at 72°C for 10 min.

### Detection of other respiratory viruses

All specimens were also screened for human respiratory syncytial virus (HRSV), human metapneumovirus (HMPV), influenza viruses A and B (IFVA, IFVB), parainfluenza virus types 1 to 3 (PIV1–3), human rhinoviruses (HRVs), and human coronaviruses (NL63 , HKU1) using a standard reverse-transcription PCR technique[16-19], and human Bocavirus (Bcov) using traditional PCR methods[20].

### Nucleotide sequence analysis

The whole hexon gene was amplified using forward primer hexon-s and reverse primer hexon-as [11,21] in our AdV11-positive specimens; the predicted product was 3449 bp. PCR was performed under the following conditions: 94°C for 1 min, followed by 35 cycles at 94°C for 30 s, 55°C for 30 s, and 68°C for 5 min, and a final extension at 68°C for 10 min.

All positive PCR products were purified using the QIAquick PCR purification kit (Qiagen), cloned into the pGEM-T Easy vector (Promega, Madison, WI, USA), and sequenced by HUADA Gene Company(Beijing, China). The nucleotide and deduced amino acid sequences of the hexon gene were compared with reference strains downloaded from GenBank. Phylogenetic analyses were conducted using MEGA software (ver. 5.05). Sequences were determined and analyzed using the DNAMAN software package .

### Statistical analysis

The statistical significance of differences between the various groups was tested using the chi-squared test and Fisher’s exact test. A *P* value of <0.05 was considered to indicate statistical significance. All analyses were performed using the SPSS software (ver. 13.0.)

## Results

### Patient characteristics

Between November 2006 and November 2009, 1169 patients were enrolled. The ages of the children with ARTIs ranged from 1 day to 168 months. The vast majority of patients (90.95%) were ≤ 5 years old. The ratio of boys to girls was 1.7:1 and that of outpatients to inpatients was 1:1.9.

### Detection of HAdVs and other respiratory viruses

In total, 74 of 1169 (6.33%) specimens were positive for HAdV. The 74 HAdV-positive cases consisted of 16 obtained in 2006–2007, 26 in 2007–2008, and 32 obtained in 2008–2009. The rate of adenovirus coinfection with other respiratory viruses was 62% (48/74); the most common coinfected virus was HRSV (15), followed by HPIV (11), Bcov (7), IFVB (5), IFVA (3), HRV (4), HMPV (4), HKU1 (1), and 2NL63 (1). Adenoviruses represented 11.68% of all Viral agents identified.

**Figure 1:**
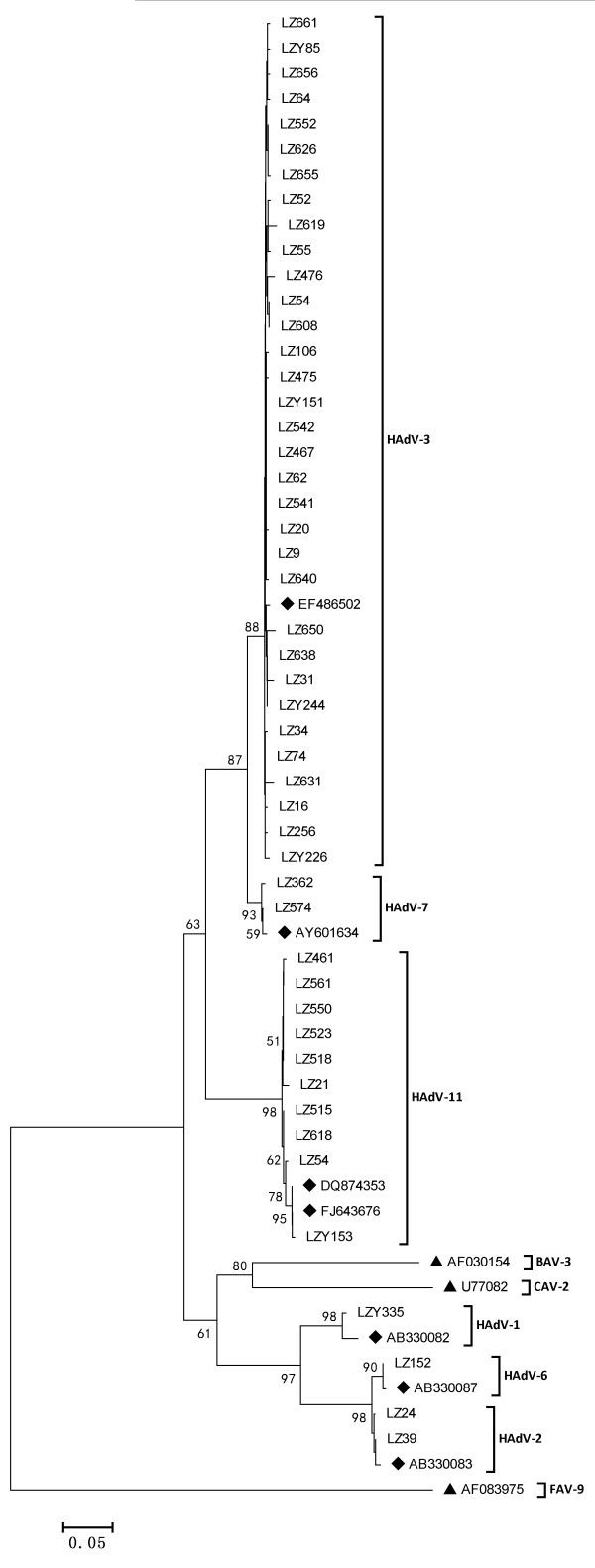
**Phylogenetic tree for Chinese ADV nucleotide sequences, based on 482 bp of the hexon gene, Phylogenetic trees were constructed by the neighbor-joining method using MEGA 5.05.** Reference strains for different HAdV genotypes, Fowl adenovirus, Canine adenovirus and Bovine adenovirus are obtained from GenBank and marked .The genotype assignment is indicated at the right by the brackets.

Of the 74 HAdV-positive cases (Figure 1), AdV3 (58/74) was detected most frequently, followed by AdV11 (10/74), AdV2 (2/74), AdV7 (2/69), AdV6 (1/74), and AdV1 (1/74). AdV3 accounted for 78.38% of HAdV positive specimens, suggesting an epidemic of ARTIs due to AdV3 during the period from November 2006 to September 2009. AdV11 was isolated sporadically throughout the study period.

### Identification of the novel HAdVs - AdV55

In total, 10 AdV11 viruses were detected in 1169 specimens, and the whole hexon gene was amplified successfully in one specimen (LZY153) collected in February 2009. The identity between LZY153 and QS-DLL (FJ643676) was 99.96%. Whole hexon gene sequence analysis showed intraspecies recombination between HAdV-11 and HAdV-14(data not show), similar to that seen for QS-DLL (FJ643676).

### HAdVs epidemiology

**Figure 2:**
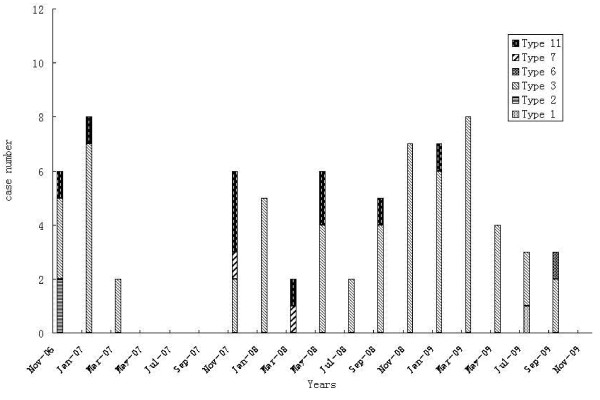
Temporal distribution of human adenovirus genotypes isolated between November 2006 and November 2009.

The monthly distribution of respiratory adenovirus infection is shown in Figure 1. No HAdV outbreak was identified in Lanzhou City, China, during 2006–2009. HAdV were detected in every month except May to October 2007, February and August 2008, and August, October, and November 2009 (Figure 2).

The mean age of the 74 AdV-positive patients was 21 (range, 0.5–144) months; 79.7% (59/74) of patients were younger than 5 years. Furthermore, 52.17% of AdV infections occurred in children <1 year of age; 11 patients were younger than 6 months, including two newborns. The peak incidence occurred at 3–4 years of age (Table 1). The ratio of males to females was 2:1.

**Table 1:** Distribution of human adenovirus (HAdVs) in different age groups

**Age (months)**	**Total cases**	**HAdVs-positive**	**% of total**	**% of positive**
0–	334	11	3.29%	14.86%
7–	184	12	6.52%	16.21%
12–	308	18	5.84%	24.32%
36–	250	25	10.00%	33.78%
>84	93	8	8.60%	10.81%
total	1169	74	6.33%	100%

### Clinical characteristics of HAdVs infection in children

Information on clinical characteristics was available for 74 patients (Table 2). Upper respiratory infection (URTI) was the most common presentation (32/74, 43.24%), followed by bronchopneumonia (21/74, 28.38%), bronchiolitis (14/74, 18.91%), and bronchitis (7/74, 9.46%). Fever was recorded in 70.27% (52/74) of the patients; prolonged (duration > 10 days) and high (temperature > 39°C) fever was recorded in 32.43% (24/74) of the patients. Cough was observed in 66.22% (49/74) of patients, wheezing in 18(58.1%), and cyanosis in two patients. Gastrointestinal (GI) discomfort, such as diarrhea, was observed in 14 (18.92%).

**Table 2:** Comparison of clinical and demographic characteristics of human adenovirus genotypes isolated between December 2006 and November 2009

**Variable**	**AdV3**	**AdV11**	**Other type**	**Total**
	**(*n*** **= 58)**	**(*n*** **= 10)**	**(*n*** **= 6)**	**(*n*** **= 74)**
Sex (male)	40 (68.97)	7 (70)	4 (66.67)	52 (70.27)
Age group (months)				
<6	8 (13.79)	3 (30)	0 (0)	11 (14.84)
6–12	7 (9.46)	4 (40)	1 (16.67)	12 (16.22)
13–24	12 (20.69)	1 (10)	0 (0)	13 (17.57)
25–60	22 (37.93)	2( 20)	2 (33.33)	26 (35.14)
>60	9 (15.52)	0 (0)	3 (50)	11 (14.86)
Mixed infections	40 (68.97)	4 (40)	1 (16.67)	45 (60.81)
Clinical manifestations				
Cough	38 (65.52)	9 (90)	2 (33.33)	49 (66.22)
Wheezing	15 (25.86)	2 (20)	1 (16.67)	18 (24.32)
Fever > 38°C	42 (70.69)	5 (50)	5 (83.33)	52 (67.57)
Crackles	21 (36.21)	7 (70)	0 (0)	28 (37.84)
Diarrhea	11 (18.97)	2 (20)	1 (16.67)	14 (18.92)
Cyanosis	2 (5.26)	0 (0)	0 (0)	2 (2.70)
Leukocyte count (median, 10^9^ leukocytes/L)	8.5	7.9		
No. of hospitalizations	27 (46.55)	8 (80)	3 (50)	38 (51.35)
Final diagnosis				
Upper respiratory tract infection	26 (44.83)	2 (20)	4 (5.41)	32 (43.24)
Bronchiolitis	11 (18.97)	3 (30)	0 (0)	14 (18.92)
Pneumonia	16 (27.59)	4 (40)	1 (1.35)	21 (28.38)
Bronchitis	5 (8.62)	1 (10)	1 (1.35)	7 (9.46)

We compared clinical characteristics between the AdV group and the RSV group (Table 3). Subjects coinfected with RSV and AdV were removed from further analysis, leaving 58 AdV-positive patients and 88 RSV-positive patients. Children infected with AdV were significantly older than children infected with RSV (chi-squared test, *p* < 0.001) and had fever more frequently (*p* = 0.030), but wheezing, cough, crackles, and cyanosis less frequently (Table 3). No significant difference in GI discomfort or the duration of hospitalization was observed between groups. But significant differences were observed in the frequency of URTI and LRTI between the two groups, and a greater frequency of LRTIs in RSV group(p < 0.001).

**Table 3:** Comparison of clinical and demographic characteristics between human adenovirus (HAdVs) and human respiratory syncytial virus (HRSV) groups in children with acute respiratory tract infections

**Variable**	**AdVs group (*n*** **= 58)**	**RSV group (*n*** **= 88)**	**p.**
Sex (male)	41 (70.69)	61 (69.32)	0.86
Median age [months; mean (range)]	42 (0.5–144)	10 (0.2–96)	<0.001
Age < 3 years	27 (46.55)	74 (84.09)	<0.001
Clinical manifestations			
Cough	34 (66.22)	83 (94.32)	<0.001
Wheezing	10 (24.32)	41 (46.59)	<0.001
Fever >38°C	42 (67.57)	48 (54.55)	0.030
Crackles	18 (37.84)	58 (65.91)	<0.001
Gastrointestinal discomfort	12 (18.92)	14 (15.91)	0.460
Cyanosis	2 (6.76)	22 (25)	0.001
No. of hospitalizations	24 (41.38)	63 (71.59)	<0.001
Final diagnosis			<0.001
Upper respiratory tract infection	30 (51.72)	2 (2.27)	
Bronchiolitis	7 (12.07)	26 (29.55)	
Pneumonia	16 (27.09)	49 (55.68)	
Bronchitis	5 (8.62)	11 (12.5)	

Chest radiographs were obtained from 18 AdV-positive patients; all showed abnormal findings. Peribronchial and perihilar infiltration were observed in 11 (11/18) of the children, coarse lung markings were observed in five (5/18), and one patient who was infected with HAdVs type 7 had pleural effusions and homogenous consolidation. Complete blood cell counts were performed for 40 of the 74 HAdVs-positive children. Most white blood cell (WBC) counts were within the normal range; for 25% (10/40) of the patients, WBC counts were >10 × 10^9^ leukocytes/L. The mean WBC count was 8.41(range, 3.3–19.9 × 10^9^ ) leukocytes/L. The overall hospitalization rate was 51.35% (38/74), with a median stay of 12.75 (range, 3–36) days.

## Discussion

In the present study, 1169 children with ARTIs were enrolled; more than half of the children (73.55%) were infected with at least one viral agent, and HAdV were detected in 74 children (6.33%), accounting for 11.68% of total virus isolates. Similar incidences of HAdV have been reported in Cuba, Taiwan, Brazil, and South Korea [1,14,22,23]. However, higher frequencies of HAdV were observed in Argentina, where rates reached 14.3% in 168 hospitalized children with LRTI during the period from 1991 through 1995[24]. HAdV infections can occur endemically throughout the year or in epidemics. Epidemics of adenoviral respiratory disease are common during the winter and spring. In the present study, although the HAdV detection rate was relatively high in winter and spring, in contrast to HRSV infections [25,26], no fixed seasonal rhythm of HAdV was identified (Figure 2).

HAdV are responsible for 4–10% of LRTIs in children [3]. Serotypes 1–3, 5, and 7 are frequently recovered from children with ARTIs, but outbreaks of severe infections in healthy children are most frequently reported with AdV7, followed by AdV3 and AdV21 [3,27,28]. In hospitalized Korean children with LRTIs due to HAdV, the serotypes recovered most frequently between 1990 and 1998 were AdV7 (41%), AdV3 (15%), and AdV2 (15%) [3]. A two-decade study from Taiwan showed that AdV3 and AdV7 were the major detected types; AdV3 was consistently isolated throughout the survey period [29]. HAdV types 3 and 7 are also two of the most important etiological agents of pneumonia in China; a previous study from China showed that AdV3 was the most prevalent genome type in children with pneumonia over the 27-year period from 1962 to 1988[27]. AdV3 and AdV7 accounted for 69–100% of HAdV strains isolated from patients with pneumonia in Beijing, China [27]. In the present study, six serotypes (types 1, 2, 3, 6, 7, 11) were observed between November 2006 and November 2009 in Lanzhou City, China. AdV3 was predominant during this period, accounting for 78.38% of HAdV. Genome recombination plays an important role in the molecular evolution of HAdV, leading to newly emerging strains that have tropism changes or become virulent [8-13,30]. Interestingly, recombination between AdV11 and AdV14 was found in our AdV11 isolates. AdV11, which has rarely been reported worldwide in ARTIs, accounted for 13.51% of HAdV isolates in our study. AdV14, which belongs to the species B2 , has been reported to cause outbreaks of severe respiratory disease in many places recently [31]. AdV11, which also belongs to subgenus B2, was isolated more frequently in China between 1965 and 1985 [32]. Since then, AdV11 infections had not been reported for more than 20 years until an acute respiratory outbreak caused by AdV55, showing a hexon recombination between AdV11 and AdV14, occurred in Shanxi Province, China, in the spring of 2008. Our study confirmed that AdV55 was present and circulating in Lanzhou, China.

A previous study indicated that the incidence of HAdV infection was inversely related to age [3]. In our study, HAdV was detected most commonly in children aged 3–7 years (10.00%), and the majority of cases (79.73%) were < 5 years old. These results are consistent with those of studies from Greater Manchester, UK, and Korea, which also revealed that most HAdV infections occurred among children less than 5 years of age [33]. However, in the present study, HAdVs infection was also found in 11 infants younger than 6 months, including two newborns. This result is in contrast to that of a previous study from Taiwan, which found that all HAdVs-positive patients except one infant were older than 6 months [34]; this phenomenon was explained by the reasoning that infants < 6 months of age are normally protected by maternal antibodies, although the exact mechanism requires further study. Our study also revealed that patients with AdV3 were significantly older than patients infected with other virus types (median age, 30 *vs*. 8 months), which is also consistent with a previous study from Taiwan. Males were more frequently infected than females with all HAdV serotypes in our study, which is also consistent with the results of a previous study [1]. All of these issues require further study.

The most common clinical diagnosis was URTI (43.24%), and 56.76% of our HAdV-positive patients had lower respiratory tract conditions, including pneumonia, bronchitis, and bronchiolitis. HAdVs were less frequent causes of ARTIs in children than were HRSV, HPIV, and HRV in our study. Furthermore, significant differences were observed in the frequency of URTI and LRTI between the RSV group and HAdV group, and a greater frequency of URTIs in HAdV group. Most LRTIs caused by HAdV are mild and indistinguishable from LRTIs caused by other respiratory viruses; however, HAdV can result in fatal outcomes or residual sequelae. In a previous study from Buenos Aires, Argentina, the overall fatality rate during hospitalization of patients with AdV7 reached 28.6%. Another study from China conducted over a 33-year period (1958–1990) reported a 15.5% fatality rate of HAdV-associated infantile pneumonia. The symptoms manifested during infection with AdV3 and AdV7 have become less severe since the mid-1980s, and no fatal case has been reported since 1986 [27]. In our 3-year study, the RTIs associated with AdV3 resulted in no mortality. This finding could be because of a stabilization of the circulating HAdV type 3 in China, with subsequent increases in herd immunity and reductions in HAdV infections. AdV7 is most frequently reported elsewhere and may be related to severe infections in healthy children [3]. In the present study, only two patients were AdV7-positive; pleural effusions and homogenous consolidation were identified on chest radiographs in one of these patients, with negative results by bacterial culturing. AdV11, which has rarely been reported in respiratory specimens, was second in frequency only to AdV3 in this study and was detected in 14.86% of AdV-positive samples. Sample LZY153, which was confirmed to contain the new AdV55 genotype with hexon recombination between AdV11 and AdV14, was collected in February 2009 from a 12-month-old boy who was admitted to the hospital because of bronchitis and eventually recovered. Given that our sample contained only one confirmed AdV55 case, the clinical significance of this genotype is difficult to analyze. Furthermore, interpretation of the general impact of HAdV infection is also restricted by the difficulty of patient follow-up for the detection of lung sequelae. Long-term surveillance of infection with different HAdV genotypes, especially newly identified genotypes, is needed to explore their clinical effects.

Of the 10 AdV-11 positive samples, only one (LZY153) was successfully amplified for the whole 3449 bp long hexon gene and was identified as AdV 55, which was assumed to be caused by the low amplification efficiency by our PCR for the long fragment and/or the degradation of the DNA in the specimens (the NPAs collected at 2006–2009 ). AdV 55 has been confirmed as a novel recombinant between AdV-11 and AdV-14 at the hexon gene [10]. And it is difficult to identify the other nine AdV11-positive samples as AdV55 by the short fragment of the hexon gene. However, this novel recombinant AdV55 needs further study to explore its clinical significance after more cases with AdV55 were reported and confirmed.

In conclusion, the present study confirmed that multiple HAdV types co-circulated in Lanzhou City, China. AdV3 predominated during November 2006 to November 2009. We also found that AdV55 existed and circulated in China. No fixed seasonal rhythm of HAdV was identified. The incidence of HAdV infection peaked in children aged 3–7 years, but a relatively high infection rate (3.29%) was observed in infants younger than 6 months, which should be given more attention. Further molecular surveillance of HAdV should be undertaken continuously to explore their epidemiological and clinical characteristics.

## Conclusions

The present study showed that HAdV is an important viral agent in children with ARTIs in Lanzhou City, China. Multiple HAdV types co-circulated with Ad3, which was predominant in this 3-year study. The novel AdV55 genotype was found in one case. No fixed seasonal rhythm could be identified.

## Competing interests

The authors declare that they have no competing interests.

## Authors’ contributions

Rong-fang Zhang carried out RT-PCR, virus detection and sequencing studies. Zhi-ping Xie, Kun-long Yan, Han-chun Gao, Jing-rong Song, Xin-hui Yuan was involved in clinical diagnosis of patients and collecting samples. Yu Jin and Rong-fang Zhang drafted the manuscript.Yu Jin, Yun-de Hou, Zhao-jun Duan conceived the study, made the arrangements for obtaining samples from the hospitals , and finalized the manuscript. All authors read and approved the final manuscript.
